# Exercise-induced desaturation during a six-minute walk test is associated with poor clinical outcomes in patients with pulmonary arterial hypertension

**DOI:** 10.1186/s40885-023-00256-3

**Published:** 2023-12-01

**Authors:** Jung Hyun Choi, Myung-Jun Shin, Byeong-Ju Lee, Jae-Hyeong Park

**Affiliations:** 1 Division of Cardiology, Department of Internal Medicine, Pusan National University Hospital, Pusan National University School of Medicine and Biomedical Research Institute, Busan, Republic of Korea; 2 Department of Rehabilitation Medicine, Pusan National University Hospital, Pusan National University School of Medicine and Biomedical Research Institute, 179 Gudeok-Ro, Seo-Gu, Busan, 49241 Republic of Korea; 3 Department of Cardiology in Internal Medicine, Chungnam National University, Chungnam National University Hospital, 282 Munhwa-Ro, Jung-Gu, Daejeon, 35015 Republic of Korea

**Keywords:** Pulmonary arterial hypertension, Desaturation, Prognosis, 6-min walk test

## Abstract

**Background:**

The six-minute walk test (6MWT) is an established exercise test for patients with pulmonary arterial hypertension (PAH), affording insight into both exercise intolerance and overall prognosis. Despite the widespread application of the 6MWT, the prognostic implications of exercise-induced desaturation (EID) during this test has been inadequately studied in PAH patients. Thus, we evaluated the occurrence of EID and its prognostic significance in PAH patients.

**Methods:**

We analyzed PAH patients in a single-center cohort from April 2016 to March 2021. EID was defined as a reduction in oxygen saturation exceeding 4% from the baseline or to below 90% at any point during the test.

**Results:**

We analyzed 20 PAH patients in this cohort, primarily consisting of 16 females with an average age of 48.4 ± 13.3 years. Among them, ten exhibited EID. Baseline characteristics, echocardiographic data and right heart catheterization data were similar between the two groups. However, total distance (354.3 ± 124.4 m vs. 485.4 ± 41.4 m, *P* = 0.019) and peak oxygen uptake (12.9 ± 3.2 mL/kg⋅min vs. 16.4 ± 3.6 mL/kg⋅min, *P* = 0.019) were significantly lower in the EID group. During the total follow-up duration of 51.9 ± 25.7 months, 17 patients had at least one adverse clinical event (2 deaths, 1 lung transplantation, and 13 hospital admissions). The presence of EID was associated with poor clinical outcome (hazard ratio = 6.099, 95% confidence interval = 1.783–20.869, *P* = 0.004).

**Conclusions:**

During the 6MWT, EID was observed in a half of PAH patients and emerged as a significant prognostic marker for adverse clinical events.

**Graphical Abstract:**

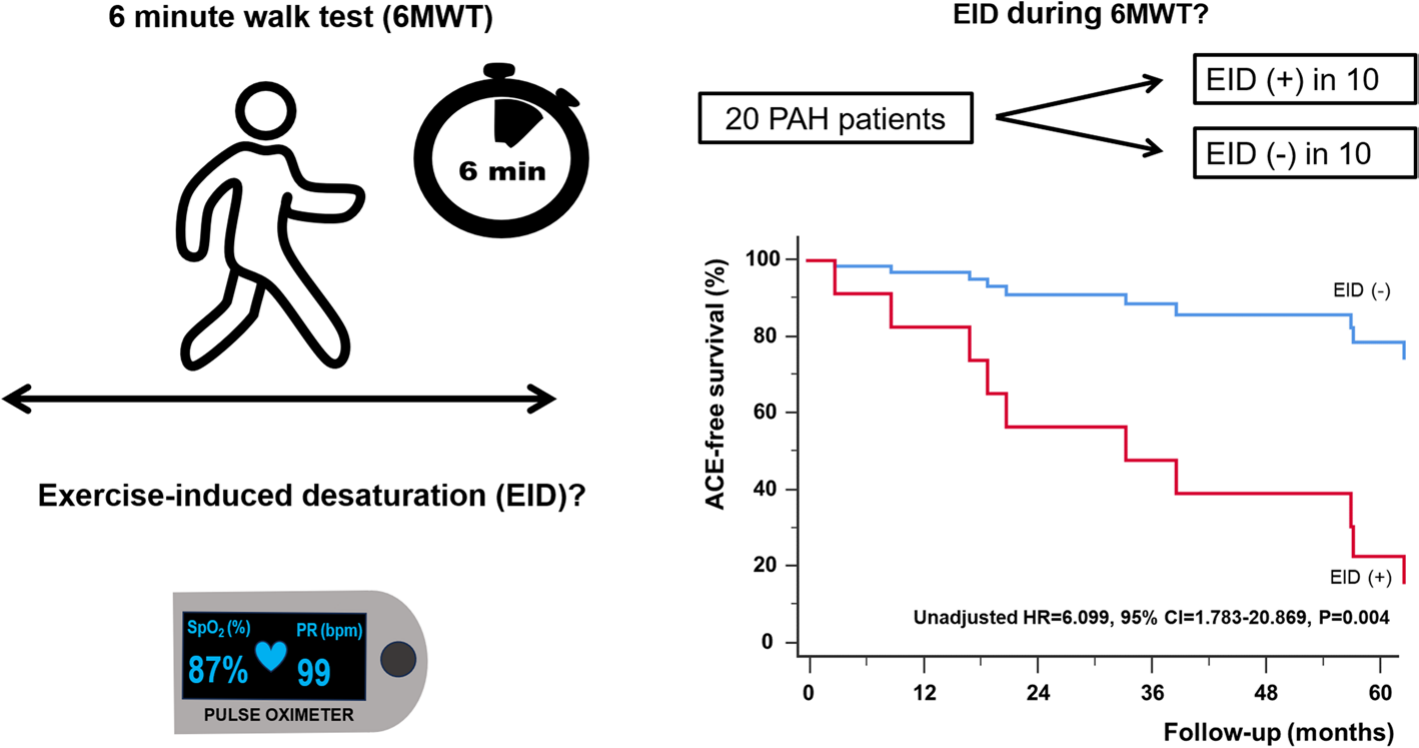

## Introduction

Pulmonary arterial hypertension (PAH) is characterized by pathological remodeling of the pulmonary vascular structures, resulting in an increase in pulmonary arterial pressure. Increased pulmonary arterial pressure leads to right ventricular (RV) failure and death, subsequently [1–3]. RV failure is common and it can be associated with worsening quality of life including progressive dyspnea, fatigue, and syncope. PAH is usually associated with decreased exercise capacity, and current treatment guidelines recommend these patients should be encouraged to engage in physical activity within their limits.

Patients with PAH may also have breathlessness due to deficient oxygenation during exercise. The degree of breathlessness can be assessed by exercise-tests. Among exercise tests, six-minute walk test (6MWT) is a simple and relatively reliable test to assess functional capacity in PAH patients [1, 2, 4]. Recent treatment guidelines for PAH recommend the use of the total distance during a 6MWT (6MWD) for the risk-stratification in the management at the diagnosis and during the treatment [1, 2].

Exercise-induced desaturation (EID) is defined as a drop in oxygen saturation (SaO_2_) of at least 4% from the baseline, or to a level of 88% or lower, during the exercise. The presence of EID is associated with increased morbidity and mortality in patients with chronic obstructive pulmonary disease (COPD) [5]. While PAH patients may show EID during 6MWT, the prevalence and importance of EID has remained relatively unexplored in the PAH population during 6MWT. Thus, we evaluated the prevalence of EID and its’ prognostic value in patients with PAH.

## Methods

### Study population

This was a single-center, retrospective, and observational cohort study of patients with pulmonary arterial hypertension (PAH) confirmed by right heart catheterization (RHC) between April 2016 and March 2021. The types of PAH were categorized according to etiologies, including idiopathic, congenital heart disease (CHD)-associated, connective tissue disease (CTD)-associated, and portopulmonary hypertension. Patients with any uncorrected CHD and suspected PAH unconfirmed by RHC were excluded. Additionally, patients in a decompensated state requiring advanced or intravenous medical therapy, patients who were not able to walk without oxygen support, and patients with other physical problems which interfered with exercise were also excluded.

The study protocol was approved by the institutional review board of the Pusan National University Hospital (approval number: H-1903–018-077). The clinical trial number is KCT0005132. This study was conducted according to the principles of the Declaration of Helsinki, and the requirement for informed consent was waived due to the retrospective nature of the research.

### Study variables and definitions

We assessed baseline demographic data and past medical history from the patients' medical records. Baseline anthropometric measurements were performed on the day of RHC. Body mass index (BMI) was calculated using height and weight with the standard formula. Hypertension was confirmed as the use of antihypertensive medication for a duration of more than 6 months, or a previous diagnosis of hypertension and being solely on lifestyle modification treatment.

Patients with diabetes mellitus (DM) were identified as those who were actively receiving treatment with oral hypoglycemic agents or insulin, or who had an abnormal fasting glucose level (≥ 126 mg/dL) or an abnormal 2-h postprandial glucose level (≥ 200 mg/dL) and were being treated with dietary modification only.

Risk assessment of PAH patients was done with the 3-strata model, low-, intermediate-, and high-risk, recommended in the current treatment guidelines depending on progression of symptoms and clinical manifestations, presence of syncope, World Health Organization (WHO) functional class, total distance at the 6MWT, cardiopulomonary exercise test, biomarkers, echocardiographic and cardiac magnetic resonance imaging data, and hemodynamic data.(Ref. 2) Because current treatment guidelines introduced in 2022, we used risk stratification model suggested by the previous guidelines [6].

We defined adverse clinical events as any of the following events that occurred during the follow-up period after the diagnosis including re-admission due to cardiopulmonary problem, lung transplantation surgery, and death.

### Echocardiographic examinations

All images were obtained using standard echocardiographic machines with standard techniques according to the American Society of Echocardiography guidelines [7]. Left ventricular (LV) end-diastolic and end-systolic volumes were calculated using the two-dimensional Simpson's method from the apical 2- and 4-chamber views, and left ventricular ejection fraction (LVEF) was calculated from these values [8]. Pulmonary arterial systolic pressure (PASP) was estimated from the peak tricuspid regurgitation jet velocity (TR Vmax) derived from the continuous wave Doppler tracing [9]. Right atrial (RA) pressure was estimated from the size and collapsibility of the inferior vena cava (IVC). RA area was calculated from the RV-focused apical 4-chamber view. Tricuspid annular plane systolic excursion (TAPSE) was measured as the distance between the end-diastolic and peak systolic points of the lateral tricuspid annulus. Tricuspid annular systolic velocity was measured at the lateral tricuspid annulus by pulsed-wave Doppler.

### Right heart catheterization

RHC was performed using a Swan-Ganz catheter via the right internal jugular vein. The mean pulmonary artery pressure (mPAP), pulmonary capillary wedge pressure (PCWP), and pulmonary vascular resistance (PVR) were measured and calculated according to the recent treatment guidelines of pulmonary hypertension.^1, 2^ Cardiac output (CO) was evaluated with a thermodilution method, and cardiac index (CI) was calculated as CO divided by body surface area.

### Pulmonary function test

A single, well-trained medical laboratory technologist administered the PFT using PONY Fx (Cosmed, Rome, Italy). All patients performed three repetitions, and the best result was accepted when the difference between each trial was within 0.150 L according to the 2017 American Thoracic Society (ATS) statement [10]. Standard spirometric measurements such as forced vital capacity, forced expiratory volume at one second (FEV1), and diffusing capacity of the lung for carbon monoxide (DLCO), were assessed.

### Six-minute walk test with gas analysis

Exercise performance was evaluated using the 6MWT with percutaneous oxygen saturation (SpO_2_) monitoring and gas analysis. We used the result of the 6MWT close to the time of diagnosis of PAH. We used the result of 6MWT near the time of the diagnosis of PAH. The exercise tests were performed when the patient was in a medically stable state just before discharge or at the outpatient clinic. 6MWT was performed on a 30-m walking track according to the ATS guidelines and observed by one skilled physical therapist [11]. SpO_2_ was observed using the wrist Ox 3150 pulse oximeter (Nonin Medical, Plymouth, MN) during the 6MWT. Using the K4b^2^ system (Cosmed, Rome, Italy), peak oxygen uptake (VO_2_peak) and the minute ventilation/carbon dioxide production (VE/VCO_2_) slope were measured simultaneously [12].

### Statistical analyses

Continuous variables are presented as means ± standard deviations, categorical variables as frequencies. For comparisons between groups, we used the Mann–Whitney U Test for continuous variables and the Fisher’s exact test for categorical variables. We used the Cox proportional hazards analysis to determine the independent predictors of adverse clinical events. Unfortunately, the study number is too small to perform the multivariate analysis because of overfitting of the model. SPSS version 25 (IBM, Chicago, Illinois, USA) was used for data analysis. A two-tailed *P* value of < 0.05 was considered statistically significant.

## Results

### Patients’ characteristics

We analyzed a total of 20 PAH patients (16 females, aged 48.4 ± 13.3 years) and summarized their baseline data in Table 1. Idiopathic PAH was the most common etiology (35%), and about 50% of patients had WHO functional class 3 and 4. At the diagnosis, 65% of patients were classified as low-risk profile.

**Table 1:** Comparison of baseline characteristics according to exercise-induced desaturation (EID)

Characteristics	Total (*n* = 20)	EID ( +) (*n* = 10)	EID (-) (*n* = 10)	*P* value
Age (year)	48.4 ± 13.3	49.7 ± 14.5	47.0 ± 12.6	0.912
Female sex (%)	16 (80)	7 (70)	9 (90)	0.582
Height (cm)	158.3 ± 7.8	158.6 ± 9.8	158.1 ± 5.6	0.912
Weight (kg)	58.1 ± 7.4	58.7 ± 9.0	57.5 ± 5.8	0.529
BMI (kg/m^2^)	23.2 ± 2.7	23.4 ± 3.7	23.0 ± 1.3	1.000
Type of PAH				0.081
Idiopathic PAH (%)	7 (35)	4 (40)	3 (30)	
CHD-associated (%)	5 (25)	0 (0)	5 (50)	
CTD-associated (%)	5 (25)	4 (40)	1 (10)	
Portopulmonary (%)	3 (15)	2 (20)	1 (10)	
WHO functional class 3 and 4 (%)	10 (50)	5 (50)	5 (50)	0.307
Risk at diagnosis				0.666
Low-risk (%)	13 (65)	6 (60)	7 (70)	
Intermediate-risk (%)	3 (15)	1 (10)	2 (20)	
High-risk (%)	4 (20)	3 (30)	1 (10)	
Cardiovascular risk factors				
Hypertension (%)	6 (30)	2 (20)	4 (40)	0.629
Diabetes mellitus (%)	2 (10)	1 (10)	1 (10)	1.000
Echocardiographic data				
LVEF (%)	57.7 ± 4.4	57.4 ± 4.5	57.9 ± 4.5	0.971
Mitral E/e’ ratio	10.2 ± 4.0	9.8 ± 3.9	10.5 ± 4.3	0.731
RVSP (mmHg)	68.8 ± 29.9	67.1 ± 27.9	70.4 ± 33.1	0.912
TAPSE (mm)	16.8 ± 4.5	16.6 ± 4.2	17.0 ± 5.0	1.000
Tricuspid annular S’ velocity (cm/s)	10.7 ± 2.8	10.5 ± 2.6	10.9 ± 3.0	1.000
RA area (cm^2^)	25.3 ± 17.6	27.3 ± 23.4	23.4 ± 9.7	0.739
Right heart catheterization data				
Mean PA pressure (mmHg)	44.0 ± 14.8	43.0 ± 10.6	45.1 ± 19.1	0.780
PCWP (mmHg)	14.7 ± 3.9	13.9 ± 2.6	15.6 ± 5.0	0.604
PVR (WU)	13.0 ± 7.9	12.0 ± 7.5	14.1 ± 8.6	0.605
CI (l/min/m^2^)	2.6 ± 0.7	2.8 ± 0.6	2.5 ± 0.8	0.340
Pulmonary function test data				
FVC (L)	2.8 ± 0.7	2.7 ± 0.9	3.0 ± 0.5	0.356
FVC (%)	80.8 ± 15.0	74.6 ± 18.1	87.7 ± 6.1	0.079
FEV1 (L)	2.2 ± 0.6	2.0 ± 0.7	2.3 ± 0.4	0.211
FEV1 (%)	75.1 ± 12.8	69.3 ± 14.7	81.4 ± 6.1	0.035
FEV1/FVC (%)	76.9 ± 7.0	75.9 ± 8.5	78.0 ± 5.3	0.447
DLCO (mL/min/mmHg)	13.6 ± 6.2	11.3 ± 4.8	16.2 ± 6.7	0.156
DLCO (%)	62.8 ± 24.4	52.7 ± 19.2	74.1 ± 25.6	0.079
6-min walk test data				
Total distance (m)	419.9 ± 112.5	354.3 ± 124.4	485.4 ± 41.4	0.019
VO_2_ peak (mL/kg⋅min)	14.6 ± 3.7	12.9 ± 3.2	16.4 ± 3.6	0.019
HR peak	118.0 ± 21.0	114.1 ± 17.8	121.9 ± 24.2	0.280
VE/VO_2_ slope (mL/min/W)	40.7 ± 12.0	44.4 ± 13.7	37.1 ± 2.9	0.247
BNP (pg/dL)	261.6 ± 290.8	414.7 ± 281.5	165.9 ± 269.2	0.284
Baseline PAH specific medication				
ERA	10 (50)	7 (70)	3 (30)	0.659
PDE5I	5 (25)	2 (20)	3 (30)	1.000
PC	6 (30)	3 (30)	3 (30)	1.000
CCB	1 (5)	0 (0)	1 (10)	0.500

*BMI* body mass index, *BNP* B-type natriuretic peptide, *CCB* calcium channel blocker, *CHD* congenital heart disease, *CI* cardiac index, *CTD* connective tissue disease, *DLCO* diffusing capacity of the lung for carbon monoxide, *ERA* endothelin receptor antagonist, *FEV1* forced expiratory volume at 1 s, *FVC* forced vital capacity, *HR* heart rate, *LVEF* left ventricular ejection fraction, *PA* pulmonary artery, *PAH* pulmonary arterial hypertension, *PC* prostacyclin, *PCWP* pulmonary capillary wedge pressure, *PDE5I* phosphodiesterase 5 inhibitor, *PVR* pulmonary vascular resistance, *RA* right atrium, *RVSP* right ventricular systolic pressure, *TAPSE* tricuspid annular plane systolic excursion, *VE* ventilatory equivalent of O_2_, *VO*_*2*_ maximal oxygen consumption, *WHO* World Health Organization

Of total 20 patients, 10 had EID during the 6MWT. Comparison of variables according to the presence of EID is presented in Table 1. There was no statistical significance of clinical, echocardiographic and RHC variables between two groups. However, EID group had significantly lower predictive percentage of FEV1 (69.3 ± 14.7% vs. 81.4 ± 6.1%, *P* = 0.035). Also, EID group had significantly lower 6MWD (354.3 ± 124.4 m vs. 485.4 ± 41.4 m, *P* = 0.019) and lower peak VO_2_ (12.9 ± 3.2 mL/kg⋅min vs. 16.4 ± 3.6 mL/kg⋅min, *P* = 0.019) during the 6MWT.

### Adverse clinical events and their determinants

**Fig. 1:**
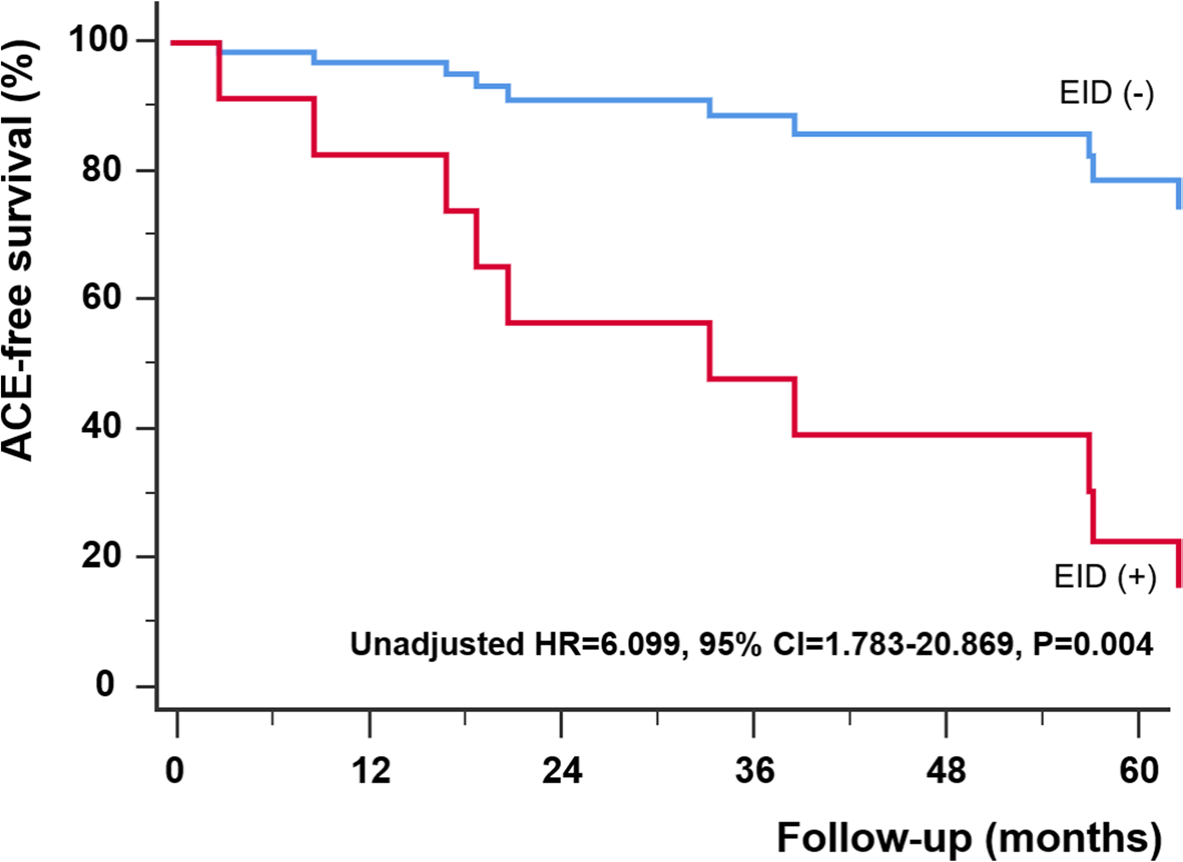
Survival analysis according to the presence of exercise-induced desaturation (EID). Patients with EID have significantly lower adverse clinical event free survival than those of patients without EID. ACE: adverse-clinical event

During the total follow-up duration of 51.9 ± 25.7 months, 17 patients had at least one adverse clinical event (2 deaths, 1 lung transplantation, and 13 hospital admissions). The results of univariate Cox-proportional hazard analysis are summarized in Table 2. Of several variables, high-risk profile at the diagnosis [hazard ratio (HR) = 4.745, 95% confidence interval (CI) = , *P* = 0.014], and 6MWD (HR = 0.992, 95% CI = 0.986–0.998, *P* = 0.009) were significantly associated with adverse clinical events. Also, EID was a significant determinant of adverse clinical event (HR = 6.099, 95% CI = 1.783–20.869, *P* = 0.004, Fig. 1).

**Table 2:** Univariate analysis for prediction of adverse clinical events

Variable	Hazard ratio	95% confidence interval	*P* value
Age (per 1 year)	1.013	0.965 – 1.062	0.604
Male sex	1.547	0.420 – 5.696	0.512
BMI (per 1 kg/m^2^)	1.250	0.966 – 1.618	0.090
WHO functional class 4	0.653	0.084 – 5.102	0.685
High-risk profile	4.745	1.362 – 16.524	0.014
Hypertension	1.869	0.606 – 5.767	0.276
Diabetes mellitus	0.495	0.064 – 3.843	0.502
LVEF (per 1%)	1.001	0.872 – 1.148	0.994
RVSP (per 1 mmHg)	1.012	0.994 – 1.031	0.179
TAPSE (per 1 mm)	0.943	0.819 – 1.085	0.410
Tricuspid annular S’ velocity (per 1 cm/s)	0.877	0.683 – 1.126	0.303
RA area (per 1 cm^2^)	1.030	0.992 – 1.069	0.120
Mean PA pressure (per 1 mmHg)	1.020	0.984 – 1.057	0.276
PCWP (per 1 mmHg)	1.025	0.904 – 1.163	0.696
PVR (per 1 WU)	1.013	0.931 – 1.103	0.761
CI (per 1 l/min/m^2^)	1.000	0.999 – 1.001	0.790
FVC (per 1 L)	0.641	0.236 – 1.745	0.384
FEV1 (per 1 L)	0.410	0.120 – 1.396	0.154
DLCO (per 1 mL/min/mmHg)	0.948	0.850 – 1.058	0.340
Total distance (per 1 m)	0.992	0.986 – 0.998	0.009
VO_2_ peak (per 1 mL/(kg⋅min))	0.861	0.712 – 1.042	0.125
HR peak (per 1/min)	0.991	0.968 – 1.015	0.465
VE/VO_2_ slope (per 1 mL/min/W)	1.039	0.992 – 1.090	0.108
EID	6.099	1.783 – 20.869	0.004

*BMI* body mass index, *CI* cardiac index, *DLCO* diffusing capacity of the lung for carbon monoxide, *EID* exercise induced desaturation, *FEV1* forced expiratory volume at 1 s, *FVC* forced vital capacity, *HR* heart rate, *LVEF* left ventricular ejection fraction, *PA* pulmonary artery, *PCWP* pulmonary capillary wedge pressure, *PVR* pulmonary vascular resistance, *RA* right atrium, *RVSP* right ventricular systolic pressure, *TAPSE* tricuspid annular plane systolic excursion, *VE* ventilatory equivalent of O_2_, *VO*_*2*_ maximal oxygen consumption, *WHO* World Health Organization

## Discussion

In this study, we found that EID was found in a half of PAH patients during a 6MWT, and it was significantly associated with poor clinical outcomes.

Exertional dyspnea and reduced exercise capacity are the main symptoms of PAH.^1^ There can be exercise-induced hypoxemia in patients with PAH, and there are several pathophysiologic mechanisms including inadequate pulmonary vascular recruitment, excessive rise in pulmonary vascular resistance resulting an insufficient increase in cardiac output of the right heart, and excessive ventilatory drive results in inadequate ventilation with high ventilatory equivalents for oxygen uptake and carbon dioxide output [13, 14]. Moreover, hypoxia can worsen pulmonary vascular resistance further by hypoxic pulmonary vascular vasoconstriction [14].

Although PAH patients have different etiology and pathologic mechanisms, PAH patients may also have shortness of breath because of deficient oxygenation during exercise like patients with other pulmonary diseases including COPD. EID has been known as a poor prognostic marker in patients with COPD. Kim et al. [15] reported that EID was a significant determinant of rapid decline of lung function in male patients with severe COPD. Liu et al. [16] showed that the presence of EID during a 6MWT was significantly associated with increased mortality in 113 COPD patients (HR = 4.12, 95% CI = 1.37–12.39, P = 0.012).

There are several studies regarding EID during a 6MWT in PAH patients. Morris et al. [17] demonstrated that EID was found more frequently in patients with CTD-associated PAH than patients with idiopathic- or CHD-associated PAH during 6MWT. They also showed that PAH patients with more severe disease and having more advanced pharmacotherapy had poorer gas exchange during a 6MWT. Like this study, we showed that EID was significantly associated with poor clinical outcomes in our study. The heightened risk of adverse clinical events, including mortality, lung transplantation, and hospital admissions, in patients with EID underscores its potential as a valuable tool for risk stratification and treatment decision-making.

In the recent data published by Ulrich et al. [14], domiciliary oxygen therapy during nights and rest at home significantly increased exercise capacity assessed by 6MWT and improved quality-of-life estimated by the SF-36 physical functioning score in PAH patients with mild resting hypoxemia and EID compared to placebo treatment. Although there has been no long-term result of oxygen therapy, domiciliary oxygen therapy can be applied in PAH patients with EID to improved their quality-of-life.

## Limitations

This study has several limitations. First, this is a retrospective cohort study from a tertiary care hospital. Also, we analyzed only 20 patients with PAH only can perform 6MWT at the time of the RHC. Because PAH is a very rare disease, the enrollment is very difficult. Thus, further studies with more study population should be needed. Second, there is difficulty in the statistical analysis. The number of patients in the study was too small, and the number of patients with events was too small to obtain an adjusted HR including other statistically significant variables. Thus, it may be difficult to determine whether the presence of EID itself predicts prognosis or whether it is influenced by other variables that differ between the two groups. In the future, a prospective study with a large number of patients from multiple centers and a well-controlled design will confirm the association between EID and its prognostic significance in PAH patients.

## Conclusions

EID can be found a half of patients with PAH during a 6MWT and is significantly associated with poor clinical outcomes. These findings have potential implications for clinical practice in PAH patients. Incorporating EID assessment during the 6MWT could enhance risk assessment and help tailor therapeutic strategies for PAH patients.

## Future perspectives

The 6MWT is a simple and widely accessible exercise test that can be easily performed in clinical settings. The identification of EID during this test could serve as an early warning sign, indicating a greater risk of poor clinical outcomes for PAH patients. Future studies could further explore the underlying mechanisms contributing to EID and its potential modulation through targeted interventions such as domiciliary oxygen therapy. Moreover, prospective investigations involving larger and diverse patient cohorts may provide additional insights into the prognostic utility of EID and its integration into the overall management of PAH patients.

## Data Availability

The datasets used and/or analyzed during the current study are available from the corresponding author on reasonable request.

## Abbreviations

6MWD: Six-minute walk distance
6MWT: Six-minute walk test
DM: Diabetes mellitus
BMI: Body mass index
CHD: Congenital heart disease
CI: Cardiac index
CO: Cardiac output
COPD: Chronic obstructive pulmonary disease
CTD: Connective tissue disease
DLCO: Diffusing capacity of the lung for carbon monoxide
EID: Exercise-induced desaturation
FEV1: Forced expiratory volume at one second
IVC: Inferior vena cava
LV: Left ventricle, left ventricular
LVEF: Left ventricular ejection fraction
mPAP: Mean pulmonary artery pressure
RA: Right atrium
RHC: Right heart catheterization
RV: Right ventricle, right ventricular
PAH: Pulmonary arterial hypertension
PASP: Pulmonary artery systolic pressure
PCWP: Pulmonary capillary wedge pressure
PVR: Pulmonary vascular resistance
SaO_2_: Oxygen saturation
TAPSE: Tricuspid annular plane systolic excursion
TR Vmax: Peak tricuspid regurgitation jet velocity
VE/VCO_2_: Minute ventilation/carbon dioxide production
VO_2_peak: Peak oxygen uptake
